# Enhancing motivation and psychological wellbeing in the workplace through conscious physical activity: Suggestions from a qualitative study examining workers' experience

**DOI:** 10.3389/fpsyg.2022.1006876

**Published:** 2022-11-24

**Authors:** Gabriele Signorini, Raffaele Scurati, Chiara D'Angelo, Marta Rigon, Pietro Luigi Invernizzi

**Affiliations:** ^1^Department of Biomedical Sciences for Health, Università Degli Studi di Milano, Milan, Italy; ^2^Department of Psychology, Catholic University of the Sacred Heart, Milan, Italy; ^3^Sports Faculty, San Antonio Catholic University of Murcia, Murcia, Spain

**Keywords:** transdisciplinary, physical literacy, ecologic approach, self-monitoring, welfare

## Abstract

**Introduction:**

After COVID-19 restrictions, hybrid solutions were established that combined smart working and work in presence. Workplace conditions significantly impact employees' lives, particularly in terms of meeting their needs and promoting their wellbeing. Based on a socio-ecological and multilevel methodology, the UP150 concept (Proactive Office 150) represents a possible innovative solution to meet employees' needs and valorize flexible work. It encourages physical exercise and active breaks during the typical workday by using particular architectural modifications, a dedicated App, and physical activity professionals as wellness coaches. The present study is the last step of the preliminary actions planned to check the benefits of the UP150 concept and aims to explore the workers' perceptions after experiencing this project.

**Methods:**

The qualitative analysis of a preliminary survey (concerning information about the company structure and workers' habits) performed before conducting a randomized controlled trial intervention study and the analysis of the semi-structured interviews after 8 weeks of a UP150 experience served as datasets for this study and have been examined and discussed.

**Results:**

In the preliminary survey, the young (under 40) and generally active (57% of the workers) reported being motivated to exercise but inhibited by a lack of time and a heavy workload. After 8 weeks at a modified workplace designed in accordance with the motive behind the UP150, the workers displayed noticeable positive perceptions and appreciation.

**Discussion:**

The qualitative analysis confirmed and supported the effectiveness of the UP150 concept that previous research had already found in quantitative parameters related to employees' motor efficiency, psychophysical status, and amount of physical activity. Participants reported beneficial perceived effects on their wellness and psychophysical status following a UP150 experience. Moreover, the concept improved social relationships and increased motivation. In conclusion, the UP150 concept efficiently fostered a positive perception of physical exercise and directed the employees toward the assumption of healthy behaviors fitting the physical literacy paradigm.

## Introduction

The COVID-19 pandemic has dramatically altered our society and human behavior. For example, the restrictions led to a change in working habits, and innovative solutions, such as smart working, have been promoted or encouraged.

The working environment is of utmost importance for people's productivity and, above all, their wellbeing. Indeed, action plans such as the Luxembourg Declaration on Workplace Health Promotion in the European Union (ENWHP, 2018) or the documents from the Italian National Institute for Public Policies' Analysis (INAPP, 2017) consider the workplace to be a fundamentally important place where people's ecologic and healthy behaviors can be altered.

The literature evinced that smart working increased the workers' sense of autonomy and independence. Research by the Nomisma observatory reported that 56% of the Italian workers interviewed would like to keep working part-time smart work after the lockdown (Nomisma, 2020).

However, even before the pandemic that negatively affected the working social context (Risi and Pronzato, 2021), an ideal office configuration was already demonstrated to positively influence autonomy, relationships between colleagues, participation in working life, and, more generally, workers' wellbeing perception (McGuire and McLaren, 2009). Furthermore, how the worker lives in the workspace is essential as well. Research on activity-based workplaces shows how getting up from the workstation and being active is beneficial. Movement-based breaks increase the workers' perception of their wellbeing as long as they do not significantly cut into productive work time (Haapakangas et al., 2018). Exercise during the usual daily workflow makes the worksite more proactive, which enhances workers' self-awareness and physical efficiency (Tsai and Wang, 2016; Jindo et al., 2019). Again, the companies benefit more from promoting employees' physical activity: productivity, work performance, and workers' mood all improve (Grimani et al., 2019), and medical costs and costs from absenteeism become considerably lower (Baicker et al., 2010).

Many organizations considered hybrid working solutions, combining smart working and working in presence during the week to meet the employees' needs (Langè and Gastaldi, 2020). This solution positively affects productivity, mental health, and work-life balance due to better management of employees' working and free time (Angelici and Profeta, 2020).

Particular attention must be paid to the worksite where the employees return to work on alternating smart-working days. The UP150 concept (Invernizzi et al., 2022) offers a possible way to meet employees' needs and valorize flexible work. UP150 stands for “proactive office,” in which 150 weekly minutes of moderate physical activity are promoted according to the World Health Organization's suggestions to preserve individual health. Specifically, the concept supports “a non-traumatic transition from the classic workplace concept (based on constriction, stress, and health risks due to a sedentary lifestyle) to a new workplace environment and office's design concept (considering the well-being and the caring of employees as central elements of companies' welfare strategies).” The UP150 concept can be defined as a theory-based intervention aiming to develop healthy habits and lifestyles from physical literacy's theoretical assumptions, contextualized and integrated into the workplace.

Physical literacy (Whitehead, 2013) starts with developing skills, knowledge, and understanding of the practiced activities, motivating physical activity, and trusting in proposals so individuals can pursue and consolidate active lifestyles and habits to preserve good psychophysical health and prevent illness. However, this assumption cannot and should not be limited exclusively to the goal of making workers healthy to preventing illnesses. Indeed, physical literacy involves a holistic, multidimensional, and broader vision of physical education that acknowledges physical exercise as having a positive impact on all dimensions (cognitive, social, affective, and motivational) of an individual (Edwards et al., 2017). Hence, to have long-term effects, the education of the movement that begins in infancy should immediately embrace the dimensions mentioned above, be continued along all evolutive stages, be retained in adulthood, and be continued until old age (Rudd et al., 2020; Carl et al., 2022).

In this monistic vision, the cognitive, psychological, social, and linked-to-movement areas are not disconnected but mutually influence each other. Indeed, moods and positive experiences combined with adequate information stimulating awareness are powerful in learning and maintaining physical abilities. Moreover, they can be more easily adapted and reused in different contexts, encompassing numerous dimensions of everyday life (Cairney et al., 2019). Since physical literacy is a holistic theory embracing multiple areas of human experience, interventions based on the previously mentioned principles need to operate in several areas to create psychophysical wellbeing by favoring “enriched” environments from both a social and structural point of view through targeted projects and adequate methodologies (Bauman et al., 2012; Lakerveld et al., 2012).

With this in mind, an increasing number of studies suggest interdisciplinary “multilevel” projects based on socio-ecological models that consider the relationship between factors that, in a given context, may affect an individual's general wellbeing more or less directly (McLeroy et al., 1988). In particular, when considering creating a favorable environment to promote psychophysical wellbeing education through motor activity, a multilevel model with different action levels should be considered, which addresses the following: (a) public policies, i.e., scientific evidence-based guidelines to disseminate in public and private institutions involved in planning and managing specific actions tailored on specific characteristics of the recipients (children, adolescents, adults, and the elderly); (b) school, corporate, and welfare-type community classifications, which should be encouraged to promote information regarding good wellbeing practices; (c) organizations involving experts prepared for designing specific interventions based on the context and recipient requirements; (d) interpersonal relationships promoting social health by increasing inclusion and productivity; (e) individual needs, i.e., focused on mental and physical health by promoting self-esteem, reducing the risks of anxiety and depression and addressing overweight and metabolic issues, respectively (McLeroy et al., 1988; Bull et al., 2020; Invernizzi et al., 2022).

The “socio-ecological filter” can also refer to the environment. It represents an essential element through which individuals can interact with a specific space that can limit (constraints) or favor (affordances) behaviors. Constraints and affordances can favor personal adaptations that benefit good wellness practices. They grant a certain degree of autonomy whenever physical exercises are directly selected and tailored according to the individual psychophysical characteristics and conditions (Bauman et al., 2012; Rudd et al., 2020). In this case, the multifunctionality and the design devoted to “spatial manipulation” are fundamental to allowing individuals to explore, discover, adapt, and self-organize their psychophysical behaviors by choosing the best response to deal with the specific individual, environmental, and task constraints affecting the different contextual situations.

UP150 principles, based on a socio-ecological and multilevel model, followed the self-determination theory's key points, including promoting autonomy, competence, and relatedness (Invernizzi et al., 2022). In this regard, the principles of self-determination theory have been widely applied in workplace design and have proven to be effective in improving employees' well-being, increasing motivation toward work and work performance (Olafsen et al., 2016; Deci et al., 2017; Rigby and Ryan, 2018). Specifically, the principles are applied *via* three main actions: architectural changes, a dedicated mobile app, and the intervention of wellness coaches (Invernizzi et al., 2022).

The present qualitative research completes a previous analysis of the effectiveness of applying the UP150 principles by Invernizzi et al. (2022). The present study aimed to investigate, through semi-structured interviews, the employees' perceptions of implementing physical activity during their working routine in a UP150 setting. Data were surveyed before, and interviews were proposed after the randomized controlled trial study by Invernizzi et al. (2022). We expect to understand which factors, among architectural changes, wellness coaches, and dedicated apps, impacted workers who alternated working at the UP150 office with smart working. We hypothesize that employees' mental and physical health improved and that these changes, whether validated by workers' perceptions or not, are the consequence of an equal contribution from the three factors.

This study explored the participants' representation of outcomes and benefits of UP150 experimentation by adding physical practice during working activity. It represents an essential asset from which a multilevel approach can be developed and maintained to promote wellness and health at work. Furthermore, the results of this study and policies that encourage cooperation between universities and factories can lead to interventions that help workers' health throughout their lives based on their needs.

## Materials and methods

### Study design

**Figure 1:**
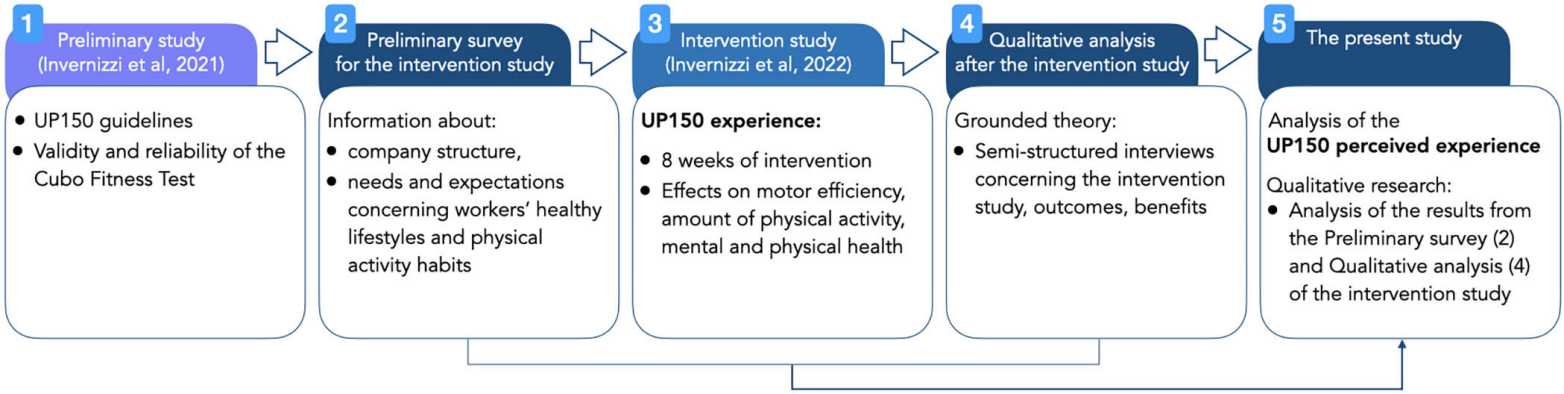
Timeline of phases and rationale of the UP150 concept and the present study's design (interventions, surveys, and analyses).

The present study completes the UP150 intervention that promoted employees' wellbeing by modeling the workplace environment to include active breaks and physical exercise during the usual daily workflow (Invernizzi et al., 2022). Figure 1 shows the timeline of the UP150 concept. The subsequent sections provide the details of each step (#1 to 5), allowing the reader to better understand the concept and its motive. Specifically, the present study analyzed the data collected before and after the experimental study of the UP150 concept (steps #2 and #4, respectively) and discussed the effects of the worker's perception of the experimental period (step #5).

#### Preliminary study

As a first step (Figure 1, step #1), a preliminary study (Invernizzi et al., 2021) was conducted on 54 individuals (21 women and 33 men, aged 20.0 ± 4.2 years). The study assessed the validity and reliability of the *Cubo Fitness Test* (CFT), the diagnostic tool designed to screen the physical efficiency of participants in the upcoming UP150 intervention research.

This phase represented the organizational setup of the concept, based on a four-step roadmap to integrate physical activity into the usual workflow: evaluating, widening, organizing, and disseminating. CFT *evaluates* motor efficiency (cardio-respiratory and muscular endurance, flexibility, core muscular efficiency, shoulder mobility, and upper body strength) through submaximal exercises. They contemplate skills involved in injury prevention (upper body strength to mitigate a fall) and promotion of lifespan functional autonomy (raising the body from a sitting/lying position to a standing one; raising the arms to reach an object placed overhead; putting on a jacket; easily brushing the back with a sponge, or flexing forward enough to put on the socks). The submaximal tests are proposed at a moderate intensity of the perceived exertion scale, and their good validity scores and reliability have already been demonstrated (Crotti et al., 2018; Invernizzi et al., 2020, 2021, 2022). *Widening* is intended to offer occasions to increase the employees' exercising during or outside of their daily working time thanks to new tools such as the UP150 App, which assists and encourages exercise and helps them arrive at a target score calculated by the app based on the quantity of movement performed weekly. Because of the CFT, the physical efficiency level can be easily established and checked. It indicates the expected weekly score to set in the UP150 App and the recommended exercise type. *Organizing* refers to managing the amount and type of physical activity to suggest to the employees based on the context and type of their job. For this purpose, professional wellness coaches guarantee the process supervision and promote the *dissemination* and activation of the whole concept.

#### Preliminary survey for the intervention study

An explorative survey (Figure 1, step #2) was conducted before the intervention study by Invernizzi et al. (2022). It was administered to the employees of a big software company (666 employees, 65% of whom were men) and encouraged and offered welfare actions based on physical activity. The survey was composed of a descriptive section in which employees were asked to report their age range, gender, and occupation and an explorative section consisting of three multiple-choice questions regarding (a) the workers' habits concerning nutrition and physical activity (one selection admitted); (b) the workers' principal motivation in engaging in physical activity (one selection admitted); and (c) possible obstacles to engaging in physical activities (multiple selections admitted). The preliminary survey's outcomes are presented in this paper's results section.

#### Intervention study

The intervention study (Figure 1, step #3) evaluated the effectiveness of the UP150 concept in increasing the participants' motor efficiency, improving psychophysical status, and increasing the amount of physical activity performed (Invernizzi et al., 2022) using the CFT, NASA-TLX, and SF-12 assessment tools, respectively and accelerometers. It involved participants from the same big software company in which the preliminary survey was accomplished.

**Figure 2:**
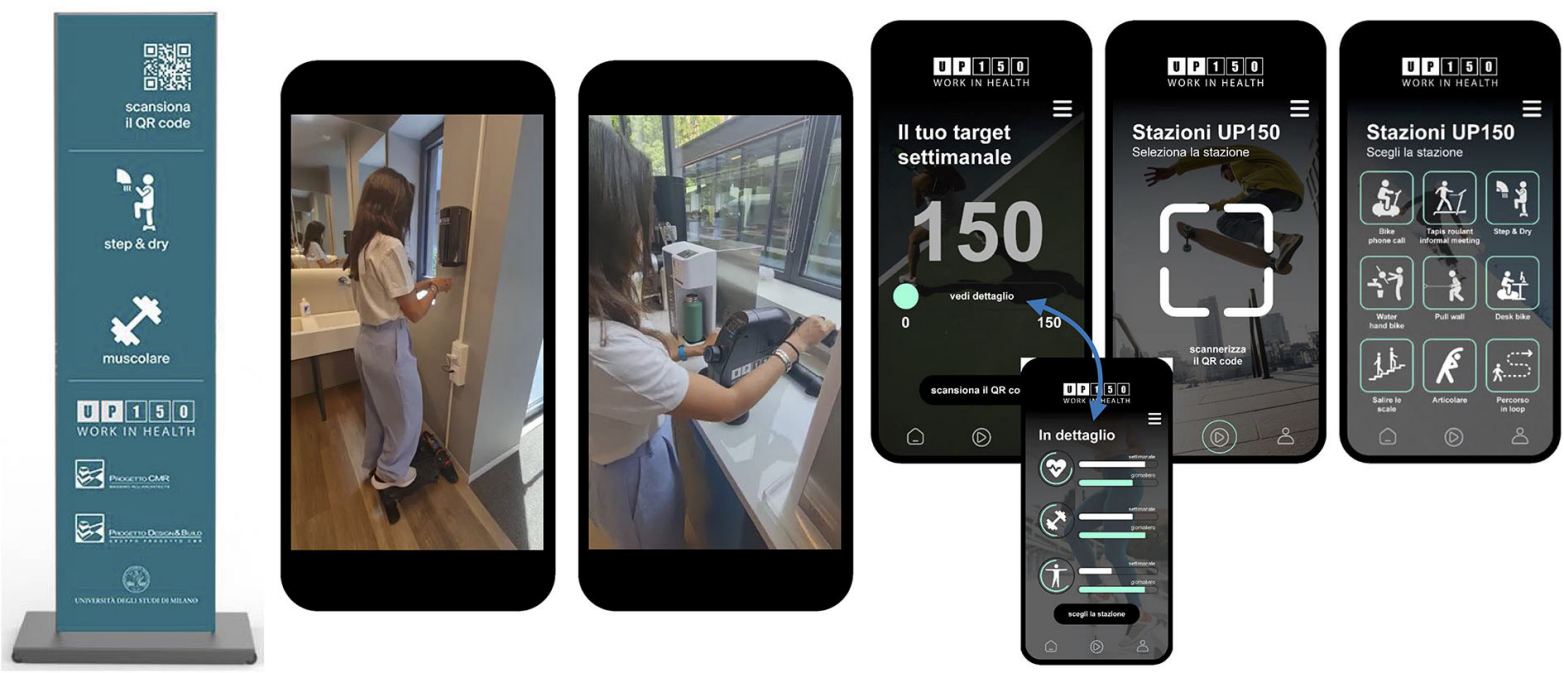
Example of architectural changes and setting for the UP150 office concept, and interaction with the UP150 App.

Forty-five volunteers were randomly assigned to an experimental group (EG, *n* = 23) and a control group (CG, *n* = 22). Five participants withdrew during experimentation and were, therefore, excluded from the study. Analyses were performed on participants who completed the protocol (EG, *n* = 20, 60% women, age 31.7 ± 8.2 years; CG, *n* = 20, 60% women, age 32.0 ± 4.4 years). EG worked for 8 weeks in the new worksite environment as designed according to the UP150 concept, alternating 3 days on-site in the office with 2 days at home in smart working. The UP150 office had been equipped with stations for active pausing or exercising during the usual workflow, which permitted strict interaction with the UP150 App by pairing fitness equipment with working and personal devices (Figure 2). Employees could access them at any time. Stations were placed to increase displacements, making different environments for short regenerating breaks without representing a source of disturbance for other employers at work. In addition, EG participants used the UP150 App to interact with architectural changes in the experimental worksite setting and to trace any physical activity performed during or outside working hours. Finally, EG participants benefited from a wellness coach who followed their physical activity and was available two times a week at the office or daily by remote video calling. In contrast, CG continued its usual working and living habits, alternating standard office work (3 days per week) with smart work (2 days per week).

In detail, architectural changes were changes that influenced the working environment. For several decades, architects have sought to integrate the fields of neurology, psychosociology, and neurophysiology into their practice. A new awareness based on scientific evidence is thus being defined for environments that have been built: they impact perceptions and, as a consequence, processes of the nervous systems, interfering with our emotions, cognitive abilities, and interactions to the point that they affect even more complex behaviors and shape our lives and actions (Higuera-Trujillo et al., 2021). The architectural changes involved not only the design of the building but even the inclusion of some physical activity stations (a treadmill in the break room, rubber bands, wooden sticks, and steppers) and the implementation of workflow physical activities (cycling or walking during online or presence meetings or the prevalent use of stairs for moving to another floor), which allowed people to interact with and live in the working space actively and innovatively. These changes required interventions on 10% of finishes (floating floors, suspended ceilings, and cladding walls), 30% of furnishings (chairs, desks), 50% of water, food, and air to dry hands, and 30% of modifications to the mechanical, electrical, and hydraulic systems. The architectural changes were also related to the use of technology to encourage autonomous and conscious approaches to physical activity within a particular context, such as the workplace.

These innovative environmental changes enabled the employee to choose what exercise to do and how much time to devote to it, favoring the use of a dedicated app over mobile devices. Indeed, all the involved employees were equipped with the UP150 App that provided a weekly activity score (a target score to reach) calculated by a system point based on activity duration and individual perceived effort (Invernizzi et al., 2021, 2022). In addition, the app also records physical activity performed during the extra-working time or the smart-working condition.

Finally, in the UP150 socio-ecological model, professional figures graduates in sports science and named wellness coaches further promoted physical activity in both presence and remote working modalities. The wellness coaches taught the employees how to practice physical fitness responsibly by self-monitoring based on the rate of perceived exertion. With their degrees, the employees were helped to reach the targeted weekly amount of physical activity and guided to correctly choose, dose, and execute the exercises during this novel experience. Self-monitoring is essential to adapt behaviors and reach psychophysical wellbeing, considering the typical socio-environmental and interpersonal situations emerging from the specific working context (presence or smart working). Assessment tools for one's psychophysical efficiency based on the perception of effort (such as the CFT by Invernizzi et al., 2021) and the employees' daily activity and physical condition self-assessment through the UP150 App (pocket trainer) allowed for self-monitoring (Suchert et al., 2013; Invernizzi et al., 2022), and aimed to boost an ever-greater competence in holding the appropriate approach to physical exercise within a work context. The employees' ability to regulate and dose physical behaviors to accommodate social work situations was promoted by a clear policy supervised by a joint academic and company and by encouraging interaction with wellness coaches that favored relationships and confrontation between employees during active breaks or breaks in dedicated spaces arranged explicitly for this purpose. The wellness coaches had to create a pleasing working environment whenever physical activity during working time was perceived as an embarrassing moment and a waste of time instead of a well-accepted opportunity for discussion among colleagues.

Altogether, these factors allowed one to choose with competence and awareness the type, place, duration, and intensity of the physical activity necessary to reach optimal psychophysical wellbeing without compromising on the assigned work. Specifically, the results of the UP150 study demonstrated an increase in physical fitness, an improvement in mental health, and a better ability to cope with the workload, highlighting the effectiveness of this multilevel approach based on promoting physical activity in supporting employees during their daily working lives.

Indeed, the motor efficiency and moderate physical activity of EG improved compared to CG. At the same time, during working activity, mental demand diminished and mental health (as measured by MCS12) increased, confirming the efficacy of the UP150 concept in acting as a proactive environment to improve the employees' physical activity, mental health, and wellness. A synthesis of the outcomes of the intervention study related to the participant's motor efficiency, psychophysical status, and increase in the amount of physical activity (Tables 1, 2).

**Table 1:** Main outcomes of the intervention study (Invernizzi et al., 2022).

**Domain**	**Tool**	**Variable**	**Group**	**Pre**	**Post**
Motor efficiency	CFT	Index of motor efficiency (AU)	EG	29.4 ± 13.7	43.0 ± 2.3^#^
			CG	32.2 ± 11.7	36.0 ± 2.6
Occupational workload	NASA-TLX	Mental demand	EG	50.3 ± 28.5	13.0 ± 24.9^#^*
			CG	40.3 ± 30.7	24.6 ± 27.0
		Effort	EG	27.2 ± 15.7	37.3 ± 24.4*
			CG	20.6 ± 17.1	60.6 ± 24.6^#^
Psycho-physical health	SF-12	MCS12	EG	37.5 ± 9.8	46.6 ± 8.2^#^
			CG	39.2 ± 9.0	42.3 ± 12.2^#^

CFT, Cubo Fitness Test; MCS12, Mental Component Summary; EG, experimental group; CG, control group; Data (scores) are shown as mean ± standard deviation. ^*^Different than CG in the between-group analysis (*p* < 0.05); ^#^Different than Pre in the within-group analysis (*p* < 0.05).

**Table 2:** Main outcomes of the intervention study (Invernizzi et al., 2022) related to the amount of physical activity measured by accelerometers at four time points (detections 1–4).

**Intensity of the physical activity**	**Detections**	**CG (min per week)**	**EG (min per week)**
Moderate	Detection 1	300.9 ± 139.0	307.8 ± 176.7
Moderate	Detection 2	281.7 ± 171.6	355.4 ± 124.8^#*^
Moderate	Detection 3	345.9 ± 200.6	392.9 ± 153.7^#§^
Moderate	Detection 4	314.7 ± 117.3	425.4 ± 175.9^#§^

Detections were performed every 2 weeks. CG, Control Group; EG, Experimental Group. Data are shown as mean ± standard deviation. *Different than CG in the between-groups analysis (*p* < 0.05); ^#^Different than detection 1 in the within-group analysis (*p* < 0.05); ^§^Different than detection 2 in the within-group analysis (*p* < 0.05).

#### Qualitative analysis after the intervention study

After 8 weeks of intervention, 20 participants (12 women and eight men, ages 31.78.2 years) in the intervention group of the study by Invernizzi et al. (2022) were interviewed (Figure 1, step #4) and composed the sample for the qualitative analysis. Specifically, the semi-structured interviews asked the following questions: (i) *What differences do you perceive in your health status at the end of the UP150 intervention?;* (ii) *Which factor among architectural changes, wellness coaches, and the training (the Cubo Fitness Test, App UP150) influenced you most? Which of these do you consider irrelevant? Is it possible to improve some of them*?; (iii) *How did you experience the integration of physical activity in the standard workflow? Underline the positive and negative aspects you perceived*; *and* (iv) *Did you perceive any change in relationships between colleagues and bosses within the working context?*

The answers were grouped to evaluate their respective frequencies. The semi-structured interviews were recorded and analyzed using the grounded theory method (Corbin and Strauss, 1990) using line-by-line coding. In the first step of open coding, themes defined as *pre-labels* were extrapolated. Next, p*re-labels* expressing similar concepts were grouped into *labels*. After that, subsequent axial coding was completed to group *labels* into *categories* that were quantified according to how often they appeared in the interviews. *Labels* with a frequency lower than 50% were defined as “sporadic,” in the 51–70% range as “typical,” and higher than 71% as “general.” The category presenting the highest percentage frequency was identified as the *core category*. This coding process was performed by two evaluators with an inter-rater consensus and successively discussed with the research team. The analysis of *labels* and *categories* was accomplished by the MAXQDA software (VERBI GmbH, 2022). The outcomes of the analysis of the interviews through grounded analysis are presented in the results.

#### Analysis of the employees' perceptions of the UP150 experience

As previously mentioned, the present study (Figure 1, step #5) ended the actions planned to assess the benefits of the UP150 concept and examined the workers' perceptions of it. The qualitative analysis of the preliminary survey (step #2, on information about the company structure and workers' habits) and semi-structured interviews after the intervention study (step #4) served as datasets to be examined and discussed.

The present study was conducted following the Declaration of Helsinki and was approved by the Ethics Committee of the University of Milan (14 September 2020, Number 84/20).

## Results

### Outcomes of the preliminary survey before the intervention study

The preliminary survey results accomplished before the UP150 intervention study depicted detailed information about the software company's employees and their habits related to healthy lifestyles.

**Figure 3:**
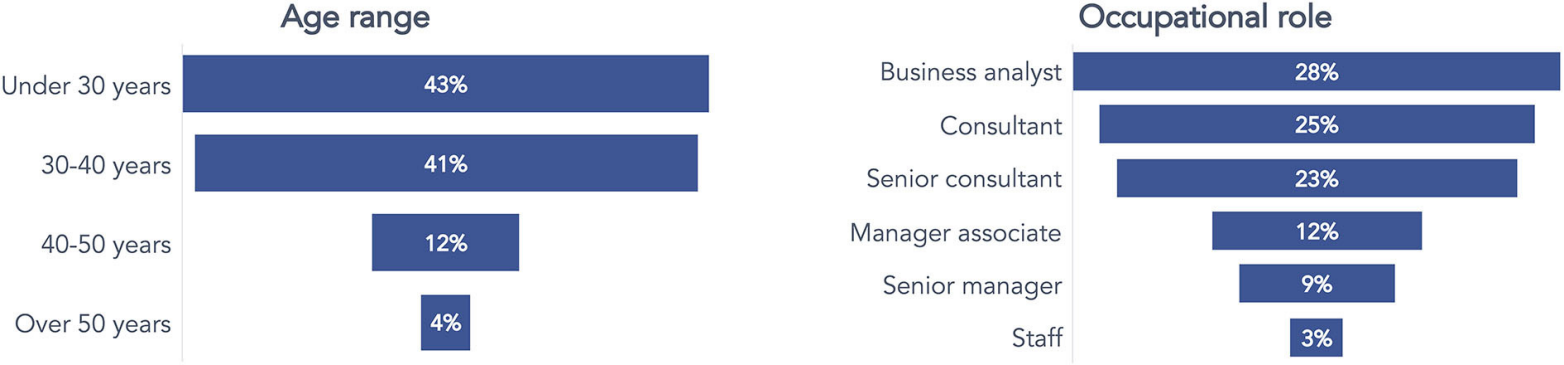
Participants' demographics concerning age and occupational role in the preliminary survey before the UP150 intervention study.

Figure 3 reports the participants' demographics by four age-range classes (under 30 years, 30–40 years, 40–50 years, and over 50 years) and their occupational roles, displaying the business setting of the company. The mean age of the employees was very low (most of them were under 30), and they predominantly worked as business analysts (28%), consultants (25%), or senior consultants (23%).

**Figure 4:**
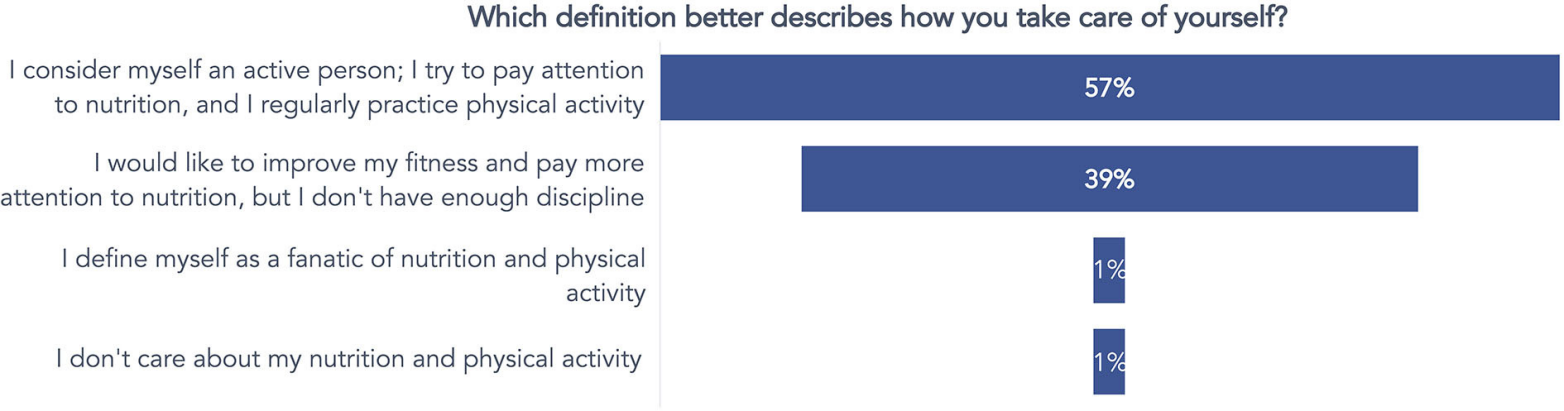
Answers to the question concerning the beliefs about personal attention to nutrition and practicing physical activity.

Three questions have been posed in the survey. The first question *(“Which definition better describes how you take care of yourself?”*) specifically investigated beliefs about nutrition and physical activity habits. As a result, most of the employees (57%) considered themselves physically active and cared about the nutritional aspects of their food. In comparison, a considerable part of the remaining respondents (39%) answered that they would like to be more active and attentive to nutrition. Still, they lack the discipline to successfully adopt and keep such a good habit (Figure 4).

**Figure 5:**
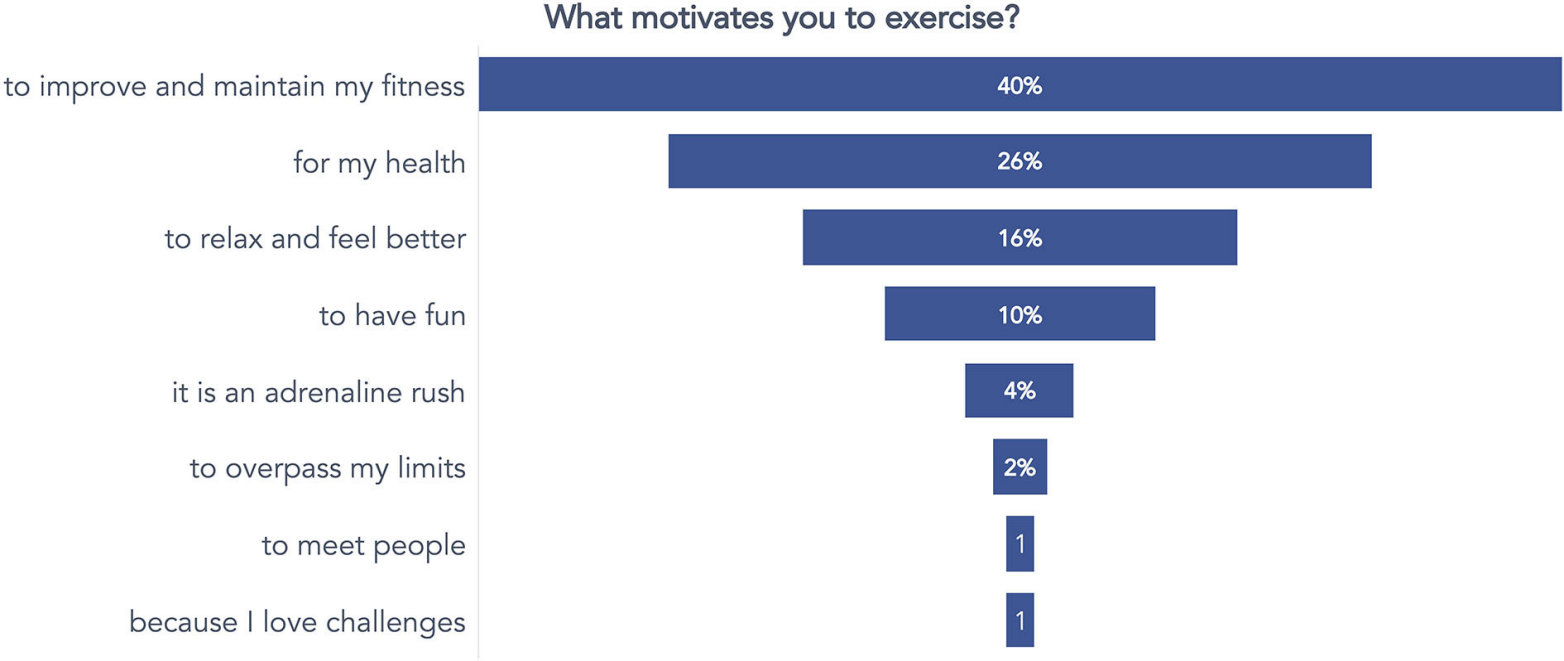
Answers to the question concerning the beliefs about motivations toward practicing physical activity.

A second question was posed to gather information about the reasons and motivations inducing respondents to engage in physical activity. Many of them (Figure 5) reported practicing physical activity to improve or maintain physical fitness (40%) and health (26%).

**Figure 6:**
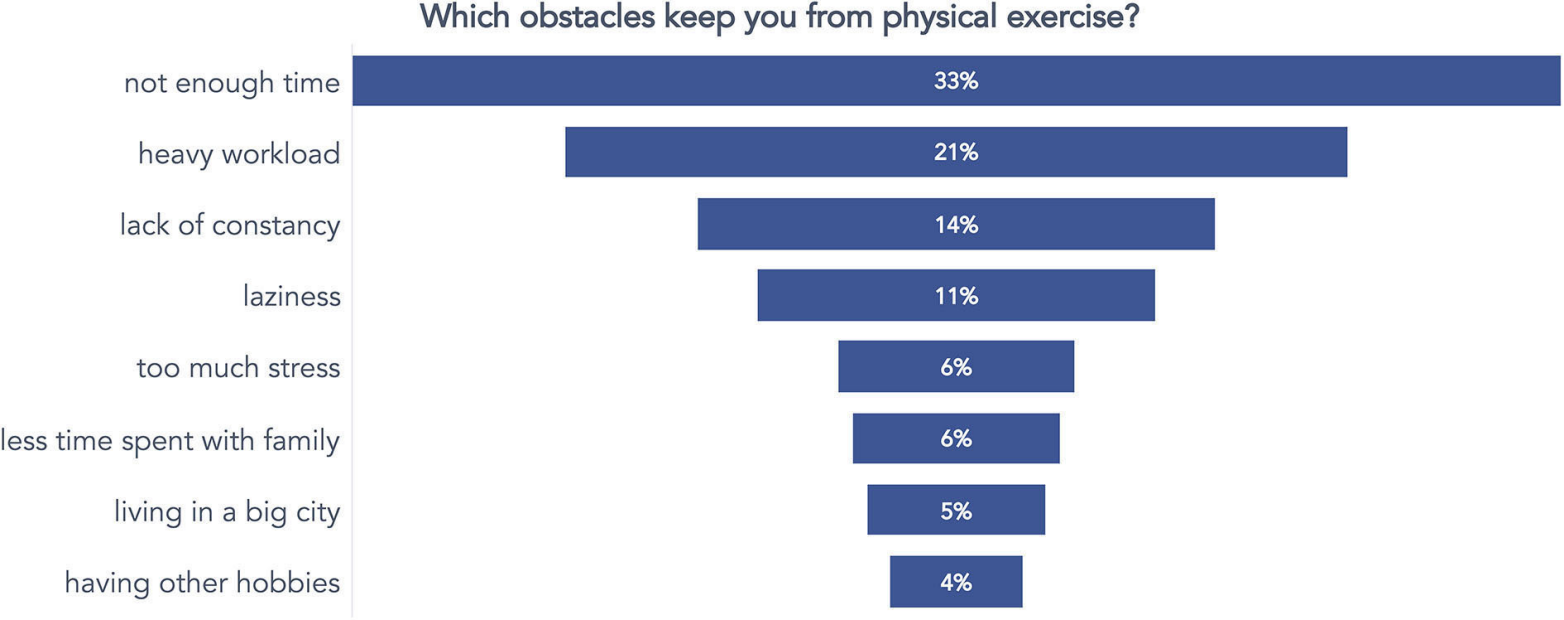
Answers to the question concerning what respondents consider a barrier to exercising.

Finally, a third question identified which exercise barriers the respondents perceived as limiting their practice. Respondents could select more than one answer. Most of them considered lack of time (33%), tiredness from daily work (heavy workload, 21%), lack of consistency (14%), and laziness (11%) as the main barriers to exercising (Figure 6).

### Outcomes of the qualitative analysis after the intervention study (analysis of the employees' perceptions of the UP150 experience)

**Figure 7:**
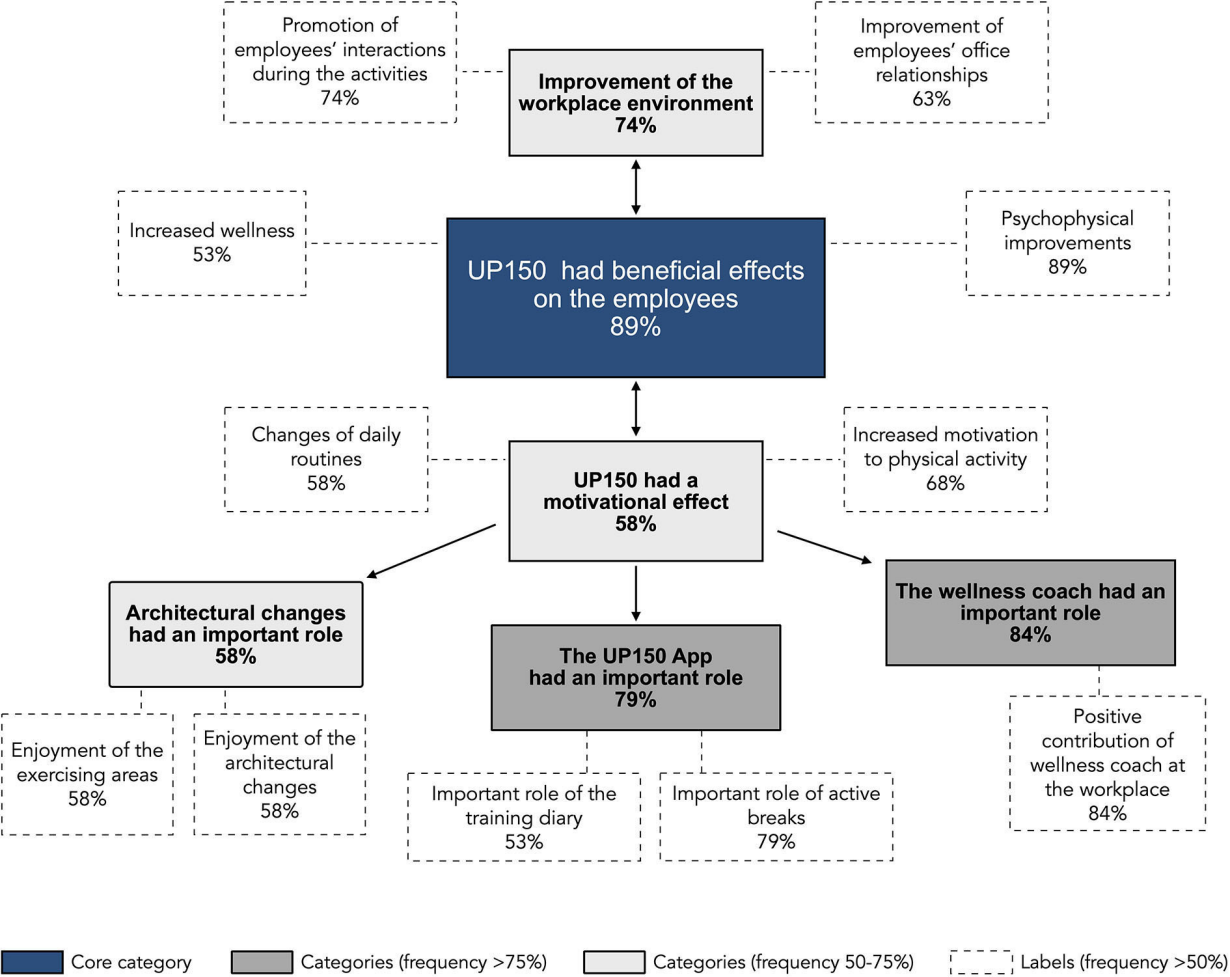
Subjects emerging from the semi-structured interview that were administered after the UP150 intervention and analyzed by the grounded theory method. The figure reports: in **blue**, the core category; in **dark gray**, the most recurrent categories (frequency of pertinent labels >75%); in **light gray**, categories having a pertinent labels occurrence between 50 and 75%; in **white**, the most recurrent labels for each respective category (which have been mentioned by at least 50% of participants).

The qualitative analysis of the semi-structured interviews conducted using the grounded theory approach allowed us to define the most recurrent subjects (categories and labels) and the connections among them (Figure 7). A core category highlighting a generally positive perception of the UP150 intervention emerged. This positive perception is associated with general wellbeing and psychophysical state improvements, as denoted by the following comments: “*I feel better while working in a UP150 context, days are lighter*,” and “*I perceived physical and mood improvements*.” In addition, while direct feedback on the overall UP150 experience was the only topic of the first question, the participants also indirectly demonstrated their satisfaction by highlighting many additional positive features in answering the other questions, which appreciation can be adduced to a junction of several further aspects.

One of them was motivation: participants reported that the intervention considerably increased their enthusiasm toward physical exercise (“*The concept encourages me to move more”*), which positively affected psychophysical wellness. In addition, the employees reported a significant change in their daily working routine in favor of a healthy-oriented lifestyle thanks to the improved predisposition to move (“*I got new and more healthy habits*”). Moreover, the element most likely affecting motivation appeared to be the presence of the wellness coach at the workplace (“*The presence of the health coach served to motivate me with constancy*”). Instead of having the wellness coaches available daily by remote video calling and just two times a week at the office, the employees would have appreciated an ulterior, more frequent attendance of them at the worksite and during worktime (“*I would have desired the wellness coaches to be more frequently available at work and during worktime*”).

The UP150 App and the architectural changes of the workplace were also suggested to have effects on motivation to exercise. Indeed, the App made the employees feel motivated to reach the targets, both at work and outside of work time (“*The App helped me be persistent in exercising through the calculation of the target score I had to reach*”). They also traced the individual physical activity performed and easily monitored the personal improvements over time (“*Thanks to the real-time update of the score, I was more conscious about the physical activity I did*”). Furthermore, the app, connected to the workplace setting, promoted frequent active breaks during the workflow, improving the workers' motivation and psychophysical wellness (“*I appreciated active breaks as they rested me from working*”). Similarly, the workplace setting has been valued by the employees, who reported they appreciated the equipment disposition and the worksite design.

Regarding socio-relational aspects, several participants conveyed that those architectural solutions improved interpersonal relationships within the business context (“*The climate at work improved: practicing physical exercise with colleagues enhanced relationships*”). Specifically, having joint active breaks or exercising helped to create more interactions and, consequently, improve communication and working relationships among colleagues (“*The workplace was comfortable, and exercising with colleagues improved relationships*”).

## Discussion

This study investigated qualitative changes in perceptions of wellness and physical engagement among employees who benefited from 8 weeks of an intervention enhancing active breaks during the working routine and daily physical exercise through architectural changes to the workplace, exercising reinforcement *via* a smartphone app, and wellness coach support. The participants, whose increased motor efficiency and mental health were retrieved in previous quantitative analyses of the intervention outcomes, benefited from the intervention and improved their perceptions of wellness and psychophysical status.

From the preliminary survey, some issues emerged and were considered to guide the upcoming intervention. For example, the prevalently young (under 40) and generally active (57%) workers reported being motivated to exercise but needed more time to do it. They were often discouraged from practicing because of the tiered workload. It is a common problem in desk workers (Desmond et al., 1993; Burton and Turrell, 2000), which drives the promotion of physical exercise in the workplace, as the UP150 concept pursues. Encouraging physical exercise in the workplace is a practical and effective solution to combat the widespread problem of sedentary behavior and accommodate the many employees who want to maintain or begin an active lifestyle but face barriers to doing so.

**Figure 8:**
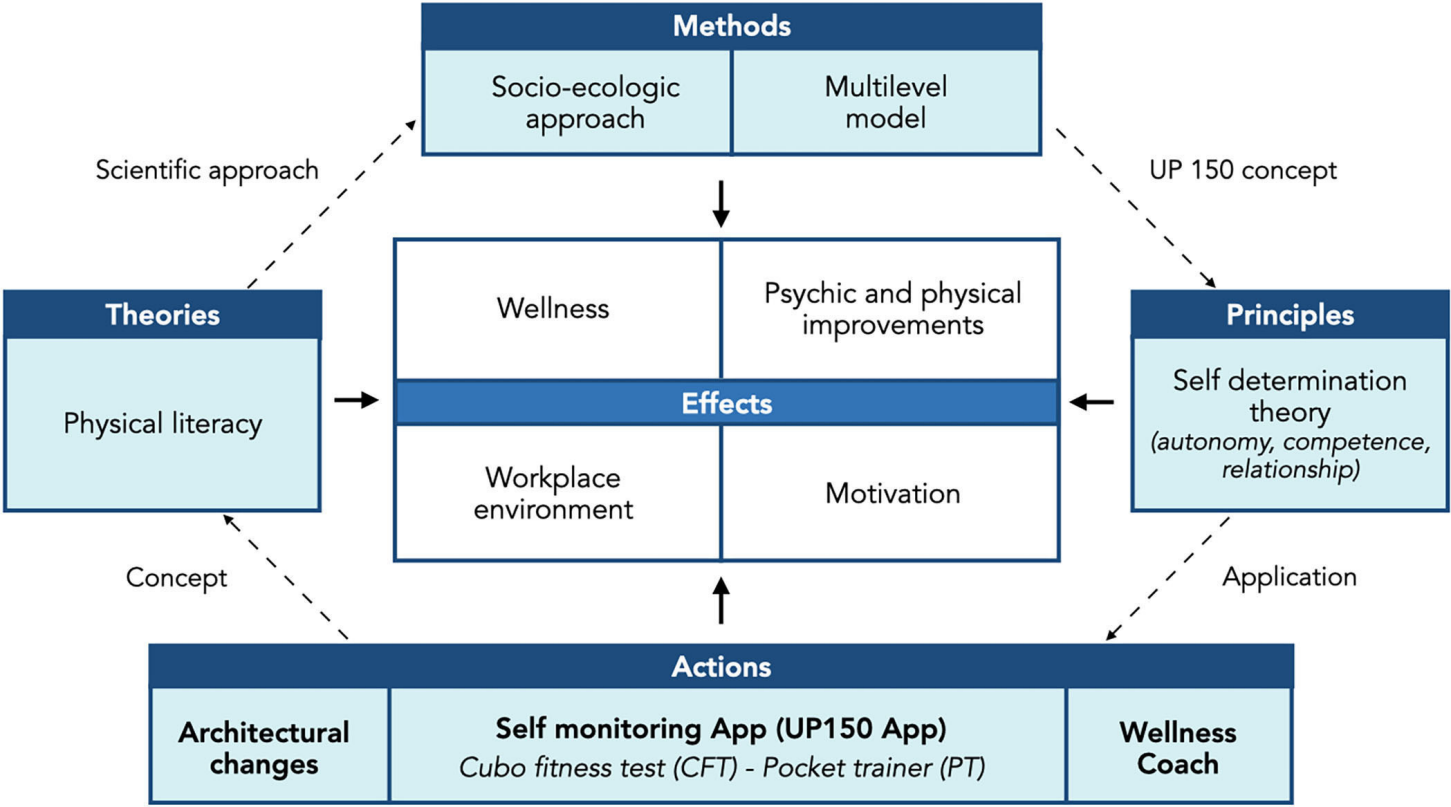
The rationale of the UP150 concept.

The workers exhibited noticeable positive attitudes and appreciated the 8 weeks in a modified workplace developed in accordance with the UP150 concept and supported by a smartphone App and wellness coaches. Specifically, from the analysis of the grounded theory model based on responses given to the semi-structured interviews, 89% of the participants in the intervention study experienced improvements in physical and mental wellbeing associated with an increase in weekly physical activity. Consistent with the intervention's multifactorial nature and relying on the principles of self-determination theory, the UP 150 concept managed components (wellness coaches, apps, and architectural modifications) to improve physical activity and engagement. As the socio-ecological model expresses, individual behaviors can modify when environmental interventions embrace different areas of social influence (McLeroy et al., 1988). In agreement with the study by Faigenbaum et al. (2020), also in the present study, the facilitators (in our case, the wellness coaches) and facilities (in our case, the architectural changes and the App) met the employees' baseline needs to overcome the barriers to exercise (workload and lack of time) that they highlighted in the preliminary survey. Figure 8 shows the UP150 concept approach and represents connections among the rationale's components, from planning to applications.

The workers' perceptions are consistent with the employees' motor efficiency, psychophysical status, and physical activity in the randomized controlled trial intervention study (Invernizzi et al., 2022). The participants improved their weekly moderate physical activity and reduced sedentary behavior, which agrees with the physical literacy concept. They urge individuals to appreciate and engage in lifelong physical activity by promoting motivation to move and mindful behaviors to reach and preserve psychophysical wellness (Carl et al., 2022). According to Rigby and Ryan (2018), the autonomy, competence, and relationship applications, which are distinctive of the self-determination theory on which UP150 stands, are the key issues enhancing motivation originating from the modified worksite that the current project provided. In this way, despite mainly being previously seen only as an impediment to healthy motor habits, the office has become a motivating and compelling part of the workers' physical activity.

The self-monitoring UP150 App further contributed to promoting physical exercise. The UP150 App merges the concepts of autonomy and competence. Due to its interactive training diary, it allowed the employees to track any performed activity and self-monitor the achievement of the weekly engagement target. Undoubtedly, the employees appreciated this support for supervising the road-to-target: “*Having a target to achieve helped me find times of day to move*,” they said. Nevertheless, such a positive tendency is frequent. It has already been studied in the literature that demonstrates the effectiveness of the hand-held devices available on the market in promoting physical exercise (King et al., 2008). Also, this innovative self-monitoring system has been designed based on perceived exertion, allowing participants to practice individually and autonomously in working and nonworking contexts. Since this underpinned self-determination principles (Deci et al., 2017), it could have fostered positive experiences and perceptions about wellness and workplace improvements, enhancing the employees' appreciation for the app.

Architectural changes, such as creating dedicated areas to favor movement and social interactions, also positively affected the company's workplace environment. The literature documents how motivational effects from workplace architecture changes can enhance job satisfaction, provided they are well contextualized and shared with the employees (Bjerke et al., 2007). In our specific case, exercising was included in the usual workflow. The percentage of workers reporting motivational effects from the UP150 concept (58%) seems to support the idea that architectural changes have been adequately contextualized in the workflow and positively shared by workers.

The appreciation of these new working areas was undoubtedly favored by wellness coaches' intervention, which is pivotal to generating competencies and promoting relationships. Indeed, the interviewees reported that their activity at the worksite had a very positive impact (“*the coaches suggested several exercises; I did not perceive shame in performing, even if I was at work*”). Nevertheless, coaches did not limit themselves to supporting the employees in exercising and adapting practices to individual needs; they had the function of sustaining employees in the transition from the standard workplace conception to a new thought about practicing physical activity in a formal environment, such as the workplace. In addition, the wellness coaches had a meaningful educational role: they guided the employees in managing the active breaks by autonomously handling exercise and training loads based on perceived exertion, thereby acquiring competence in self-monitoring (Butterworth et al., 2006; Suchert et al., 2013).

Notably, active breaks during the usual workflow have been appreciated by 79% of participants. The introduction of such a strategy was fundamental for enhancing the employees' motivation to exercise and helped the wellness coaches guide employees' attention and techniques for managing workloads. Underneath such strategies, active breaks (and ways to address them) enabled autonomy, competence, and relationships. They appropriately responded to one of the occurrences that emerged from the preliminary survey (Figure 6), impeding or limiting the practice of physical activity: a lack of time and “work pressures,” often causing anxiety and extra-working engagements. As previous research already highlighted (Invernizzi et al., 2022), the active breaks strategy allowed workers to find time to exercise at work and offered an approach to dealing with working loads and mental stress (Teixeira et al., 2012).

Including exercises in the workflow exploited the workers' routines: “*I have found a place for new routines throughout my working day*.” Further habits and adaptations to the modified environment originated from the new workplace concept (and, therefore, from the alternative idea of workflow). These healthy habits fit the reference theory on which this study is based, i.e., physical literacy. Hence, the UP150 concept created a framework also permitting the workplace to become an educational place, promoting healthy habits that workers can preserve until they are elderly, and allowing workers to manage their working time and workloads adequately.

The socio-ecologic approach positively affected the intrapersonal sphere: participants reported improvements in the company's social climate. These improvements have been attributed to the active-break strategy and the advancement of active relationship occasions. Indeed, in the socio-ecologic model, the social environment is decisive in advancing physical exercise: watching other people being active or practicing physical activity at an organizational level is one of the factors contributing to physical exercise promotion (Bauman et al., 2012). In the UP150 concept, active breaks and interactions were intended to make the employees interact during the workday and enhance interpersonal relationships. Positive benefits from the company's workplace environment are not unexpected: wellness at work highly depends on the working environment, which further relies on workers' interpersonal relationships (Desrumaux et al., 2015). The UP150 concept, in particular, promoted a positive company culture by encouraging employees to interact with one another through the use of active breaks within a socio-ecologic multilevel-model framework, which in turn influenced the employees' perception of the intervention's positive effects on their health (Invernizzi et al., 2022). The improved mental health of the experimental group (workers in the UP150 office) in comparison to the control group (employees in a conventional workplace) confirmed the coping impact of physical exercise in high-position employees (Wandel and Roos, 2005; Faulkner et al., 2020).

The company's peculiarities and the limited duration of the intervention represent some limitations. Indeed, the intervention was conducted in an IT company with specific features that may differ from other workplaces (e.g., the employees' mean age or educational status). This could have affected the results, making it necessary to recommend extending further research to companies with different objectives and organizational charts. In addition, the results were from 8 weeks of intervention, a relatively short time. Even if the results howed a clear positive trend in changing behaviors and perceptions, longer interventions might strengthen the effectiveness of the UP150 concept. Longitudinal studies in different workplaces must confirm and generalize the positive effects on behavior and perception and their long-term preservation.

## Conclusion

The qualitative analysis of the perception of workers' UP150 experience in the present study confirmed and supported the efficacy of the new-concept worksite office previously investigated by Invernizzi et al. (2022) as effective for workers' wellbeing, possibly further favoring the return to work at the office after an extended period of remote working after the COVID-19 pandemic. Precisely, an enriched working environment can be created by providing the following: (i) architectural changes, the UP150 App, and wellness coaches' interventions; (ii) a multilevel- and socio-ecologic-based approach, and (iii) the application of the self-determination principles. These actions can efficiently promote physical exercise and address the assumption of healthy behaviors that fit the physical literacy paradigm.

## Data availability statement

The raw data supporting the conclusions of this article will be made available by request to the authors, without undue reservation.

## Ethics statement

The studies involving human participants were reviewed and approved by the Ethics Committee of the University of Milan (14 September 2020, Number 84/20). The patients/participants provided their written informed consent to participate in this study.

## Author contributions

GS and PI contributed to conception and design of the study. CD'A supervised methodology. MR and GS organized the database. CD'A and GS performed the formal analysis. GS, PI, and RS wrote the first draft of the manuscript. PI and RS supervised the study. All authors contributed to manuscript revision, read, and approved the submitted version.

## Conflict of interest

The authors declare that the research was conducted in the absence of any commercial or financial relationships that could be construed as a potential conflict of interest. The reviewer NL declared a shared parent affiliation with the authors GS, RS, MR, and PI to the handling editor at the time of review.

## Publisher's note

All claims expressed in this article are solely those of the authors and do not necessarily represent those of their affiliated organizations, or those of the publisher, the editors and the reviewers. Any product that may be evaluated in this article, or claim that may be made by its manufacturer, is not guaranteed or endorsed by the publisher.
